# Creatinine-lactate score predicts mortality in non-acetaminophen-induced acute liver failure in patients listed for liver transplantation

**DOI:** 10.1186/s12876-021-01830-5

**Published:** 2021-06-07

**Authors:** Estela Regina Ramos Figueira, Joel Avancini Rocha-Filho, Cinthia Lanchotte, Lucas Souto Nacif, Luciana Bertocco de Paiva Haddad, Adriana Rochetto Assalin, Yumi Ricucci Shinkado, Agustin Moscoso Vintimilla, Flavio Henrique Ferreira Galvao, Luiz Augusto Carneiro D’Albuquerque

**Affiliations:** 1 Divisão de Cirurgia do Aparelho Digestivo, Departamento de Gastroenterologia, Hospital das Clinicas da Faculdade de Medicina da Universidade de Sao Paulo - HCFMUSP, Av. Dr. Arnaldo 455, 3rd floor, office 3222, Sao Paulo, SP Zip code: 01246-903 Brazil; 2 Laboratorio de Investigaçao Medica 37, Departamento de Gastroenterologia, Hospital das Clinicas da Faculdade de Medicina da Universidade de Sao Paulo - HCFMUSP, Sao Paulo, SP Brazil; 3 Disciplina de Anestesiologia, Hospital das Clinicas da Faculdade de Medicina da Universidade de Sao Paulo - HCFMUSP, Sao Paulo, SP Brazil; 4 Serviço de Transplante de Figado e Orgaos do Aparelho Digestivo, Departamento de Gastroenterologia, Hospital das Clinicas da Faculdade de Medicina da Universidade de Sao Paulo - HCFMUSP, Sao Paulo, SP Brazil; 5 Faculdade de Medicina da Universidade de Sao Paulo - FMUSP, Sao Paulo, SP Brazil

**Keywords:** Acute liver failure, Bilirubin, Creatinine, Lactate, Prognosis

## Abstract

**Background:**

The aim of this study was to analyze prognostic indicators of in-hospital mortality among patients listed for urgent liver transplantation (LT) for non-acetaminophen (APAP)-induced acute liver failure (ALF).

**Methods:**

ALF patients listed for LT according to the King’s College Criteria were retrospectively reviewed. Variables were recorded from medical records and electronic databases (HCMED and RedCap).

**Results:**

The study included 100 patients, of which 69 were subject to LT and 31 died while waiting for LT. Patients were 35.5 ± 14.73 years old, and 78% were females. The main etiologies were virus (17%), drug-induced (32%), autoimmune (15%), and indeterminate hepatitis (31%). The prioritization-to-LT time interval was 1.5 days (0–9). The non-LT patients showed higher lactate (8.71 ± 5.36 vs. 4.48 ± 3.33 mmol/L), creatinine (229 ± 207 vs. 137 ± 136 µm/L), MELD (44 ± 8 vs. 38 ± 8), and BiLE scores (15.8 ± 5.5 vs. 10.3 ± 4.1) compared to LT patients (p < 0.05). Multiple logistic regression analysis identified creatinine and lactate as independent prognostic factors, and a creatinine-lactate (CL) score was developed. ROC analysis showed that creatinine, lactate, MELD, BiLE, and CL scores had considerable specificity (71–88%), but only BiLE, lactate, and CL presented high sensitivities (70%, 80%, and 87% respectively). AUCs were 0.696 for creatinine, 0.763 for lactate, 0.697 for MELD, 0.814 for BiLE, and 0.835 for CL.

**Conclusions:**

CL and BiLE scores predict mortality with more accuracy than MELD in patients with ALF during prioritization time. Creatinine and lactate are independent prognostic factors for mortality.

## Background

The mortality of non-acetaminophen (APAP)-induced ALF patients listed for liver transplantation is an important concern. Acute liver failure (ALF) is a life-threatening multisystem syndrome that is defined by the development of encephalopathy during the course of jaundice in patients without previous liver disease [1]. The development of this syndrome is unpredictable, and the rate of spontaneous recovery is less than 20% in non-APAP patients [2].

In the last decades, the introduction of routine liver transplantation (LT) as a treatment option for ALF patients with poor prognosis has been a turning point, and the survival rate has increased dramatically [3]. Since then, several authors have proposed prognostic criteria to indicate LT for ALF patients with little chance of spontaneous recovery. In this setting, the King’s College Criteria (KCC) [4] are largely used worldwide to select patients with less than 15% probability of survival without LT. On the other hand, the KCC have low sensitivity and do not discriminate disease severity after LT indication, so some patients who are likely to die are not identified [5].

A great number of ALF patients still fail to receive transplants and die while waiting for a graft [6]. Furthermore, the KCC and others, such as the Clichy criteria [7], have been criticized among transplant surgeons for indicating LT too late in some patients who are too sick. During the waiting period for LT, progression of the disease can lead to serious clinical complications, including the development of coma due to increased cerebral edema, renal failure, systemic inflammatory response syndrome, sepsis, and multiple organ failure [8, 9]. These states directly affect transplantation outcomes, thereby reducing the survival rate. Thus, decreasing patient death during the waiting period for transplantation is crucial to improve survival [10].

In a multicenter study including 267 patients with ALF, Escorsell et al. [3] observed very poor prognosis among patients with transplant contraindication in fulfilling KCC. Although 11 deaths (6.8%) had occurred on the waiting list, there were no proposals to increase the chance of LT for these patients. Another report suggests that the Model for End-Stage Liver Disease (MELD) classifies ALF patients with higher scores that are associated with greater benefit from LT [11].

Some authors show that poor outcome after LT is correlated with renal function, acidosis, and severity illness indexes such as Apache III [12]. Nevertheless, contraindication for LT remains controversial. The final decision for LT is still highly dependent on the subjective evaluation based on the transplant team’s personal experience [13]. Furthermore, there are no objective and sufficiently discriminatory parameters that can properly select patients with disease progression and those who are too sick to undergo transplantation. Therefore, in the present study, we aimed to analyze prognostic indicators of in-hospital mortality among patients listed for urgent LT for acute liver failure.

## Methods

### Patients

We retrospectively analyzed 100 patients with ALF who were referred for urgent LT and were admitted to the Liver and Gastrointestinal Transplant Division of Hospital das Clinicas, University of Sao Paulo School of Medicine. The study was approved by the Ethics Committee of the Hospital das Clinicas (CAPPesq-HCFMUSP number 4245/2012). ALF was defined according to Trey and Davidson’s criteria [1], which consider the 8-week interval between the onset of jaundice and the development of encephalopathy in the absence of previous liver disease. All patients enrolled in this study had indication for LT according to the KCC, which is defined by the presence of an international normalized ratio of prothrombin (INR) greater than 6.5 or at least 3 of the following five variables: (A) interval jaundice/encephalopathy greater than 7 days, (B) age less than 10 or greater than 40 years, (C) indeterminate etiology or drug-induced hepatitis, (D) total serum bilirubin greater than 300 μmol/L, and (E) INR greater than 3.5 [4].

During the preoperative period, patients were subjected to a standard care protocol of preemptive cefotaxime **(**2 g t.i.d.) and fluconazole (400 mg q.d.). Patients presenting hepatic encephalopathy grade 3 or 4 according to the West-Haven criteria [14, 15] were electively intubated for mechanical ventilation. All patients were monitored for hemodynamic instability and the occurrence of cerebral edema. Treatment for cerebral edema was based on a 30° head-of-bed elevation, restriction of intravenous hydration according to the intravenous central pressure, and correction of hypoxemia and/or hypercapnia. In selected cases, an epidural catheter was placed to monitor intracranial pressure. Hemodialysis or continuous venous-venous hemofiltration was initiated when indicated.

### Study variables

Clinical and laboratory data were recorded from medical records and our electronic databases HCMED and RedCap. All variables were recorded from day 1 of the patient listing for LT. The following parameters were recorded: (1) clinical parameters: age, sex, etiology of hepatitis, jaundice-to-encephalopathy time interval, grade of hepatic encephalopathy (1–4), time interval from patient listing to transplantation (if applicable), date of liver transplantation, date of death (if applicable); (2) laboratory data: INR, total serum bilirubin, serum creatinine, arterial blood lactate, serum alanine aminotransferase (ALT), serum aspartate aminotransferase (AST), MELD score [16], and the bilirubin-lactate-etiology (BiLE) score [17]. The following scores were calculated:

$${\text{MELD}}\,{\text{score}}\, = \,{9}.{57}\, \times \,{\text{Ln}}\left( {{\text{creatinine}}\,\left[ {\text{mg/dL}} \right]} \right)\, + \,{3}.{78}\, \times \,{\text{Ln}}\left( {{\text{bilirubin}}\,\left[ {\text{mg/dL}} \right]} \right)\, + \,{11}.{2}0\, \times \,{\text{Ln}}\left( {{\text{INR}}} \right)\, + \,{6}.{43}\quad \quad \,\left[ {{16}} \right]$$

$${\text{BiLE}}\,{\text{score}}\, = \,{\text{bilirubin}}\,\left( {\upmu {\text{mol/L/1}}00} \right)\, + \,{\text{lactate}}\,\left( {\text{mmol/L}} \right)\, + \,{4}\,\left( {{\text{if}}\,{\text{indeterminate}}\,{\text{ALF}}\,{\text{or}}\,{\text{Budd}}{-}{\text{Chiari}}\,{\text{syndrome}}} \right) \quad \left[ {{17}} \right]$$

Additionally, a new creatinine-lactate (CL) score was mathematically developed using logistic regression and calculated as:

$$0.{3694}\, \times \,{\text{creatinine}}\,\left( {\upmu {\text{mol/L}}} \right)\, + \,{24}.{1928}\, \times \,{\text{lactate}}\,\left( {\text{mmol/L}} \right)$$

### Statistical analysis

The primary end point of this study was death due to any cause during pre-transplant hospital stay. Results were expressed as the means with standard deviations, medians with ranges, and percentages in some cases. Categorical variables were evaluated with the Fisher exact test. Continuous variables were evaluated with a *t* test or the Mann–Whitney U test according to the normal distribution of variables. Simple and multiple logistic regressions were performed to determine the prognostic value. ROC curves were calculated to complete the evaluation of the most important variables. Statistical analyses were performed with R Statistical Software for Windows, version 2.12.

## Results

### Demographic characteristics and clinical data

Among the 100 patients referred for liver transplantation for ALF, the mean age was 35.5 ± 14.7 years, and 78% were women. The etiologies were viruses in 17% of cases, drug-induced liver injury (DILI) in 32%, autoimmune in 15%, indeterminate in 31%, and other causes in 5%. The prioritization-to-LT time interval was 1.5 (0–9) days, and the grade of encephalopathy at prioritization was 3 (1–4). The median interval between the development of jaundice and the onset of encephalopathy was 15 (1–60) days in the whole group, with 15 (1–60) days in the group of transplanted patients and 14 (1–60) days among patients who died before transplantation. Multiple organ failure associated with ALF was the main cause of death in non-transplanted patients.

A comparison of clinical and biochemical features at baseline between both groups is summarized in Table 1. The univariate analysis suggested that indeterminate and autoimmune etiologies, creatinine, lactate, and MELD, BiLE, and CL scores were different between groups (Table 1). However, there were no differences in clinical characteristics such as age, sex, encephalopathy grades, the time to development of encephalopathy after onset of jaundice, and the waiting list time, or in laboratory variables such as INR, AST, and bilirubin.

**Table 1:** Patients’ characteristics at prioritization according to outcome

Variables	LT group n = 69	Non-LT group n = 31	*p* value
Age: mean ± SD, y	34.5 ± 13.7	37.7 ± 16.8	0.5000
Sex women: n (%)	51 (73.9)	27 (87.1)	0.1937
*Encephalopathy: n (%)*			
Grades I/II	10 (14.7)	10 (32.2)	0.0592
Grade III/IV	58 (85.3)	21 (67.8)	
*Etiology: n (%)*			
A or B or C virus	13 (18.8)	4 (12.9)	0.3368
DILI	21 (30.4)	11 (35.5)	0.6479
Autoimmune hepatitis	14 (20.3)	1 (3.2)*	0.0212
Indeterminate	17 (24.6)	14 (45.2)*	0.0358
Wilson’s disease	2 (2.9)	2 (6.5)	0.5858
Budd-Chiari syndrome	1 (1.5)	0	1.0000
*Jaundice-encephalopathy interval: n (%)*			
≤ 7 days	18 (28.1)	4 (16.0)	0.1803
> 7 and ≤ 28 days	28 (43.8)	13 (52.0)	0.3204
> 28 days	18 (28.1)	8 (32.0)	0.7970
Waiting list time: median (min–max), days	1 (0–9)	2 (0–7)	0.5020
INR: mean ± SD	5.09 ± 2.77	6.47 ± 3.90	0.1600
AST: mean ± SD, U/L	578 ± 578	493 ± 779	0.0590
Bilirubin: mean ± SD, mg/dL	28.31 ± 10.06	29.34 ± 11.44	0.6690
Creatinine: mean ± SD, mg/dL	1.55 ± 1.54	2.59 ± 2.34*	0.0020
Lactate: mean ± SD, mg/dL	40.35 ± 29.28	78.43 ± 48.27*	< 0.0001
MELD	38.3 ± 8.0	44.1 ± 8.0*	0.0010
BiLE score	10.3 ± 4.1	15.8 ± 5.5*	< 0.0001
Creatinine-Lactate score	160.4 ± 94.8	296.1 ± 131.0*	< 0.0001

LT group, liver transplant group; Non-LT group, patients died before LT; DILI, drug induced live injury; MELD, model for end-stage liver disease; BiLE score, bilirubin lactate etiology score; *p < 0.05 compared to LT group

### Etiology of acute liver failure

**Fig. 1:**
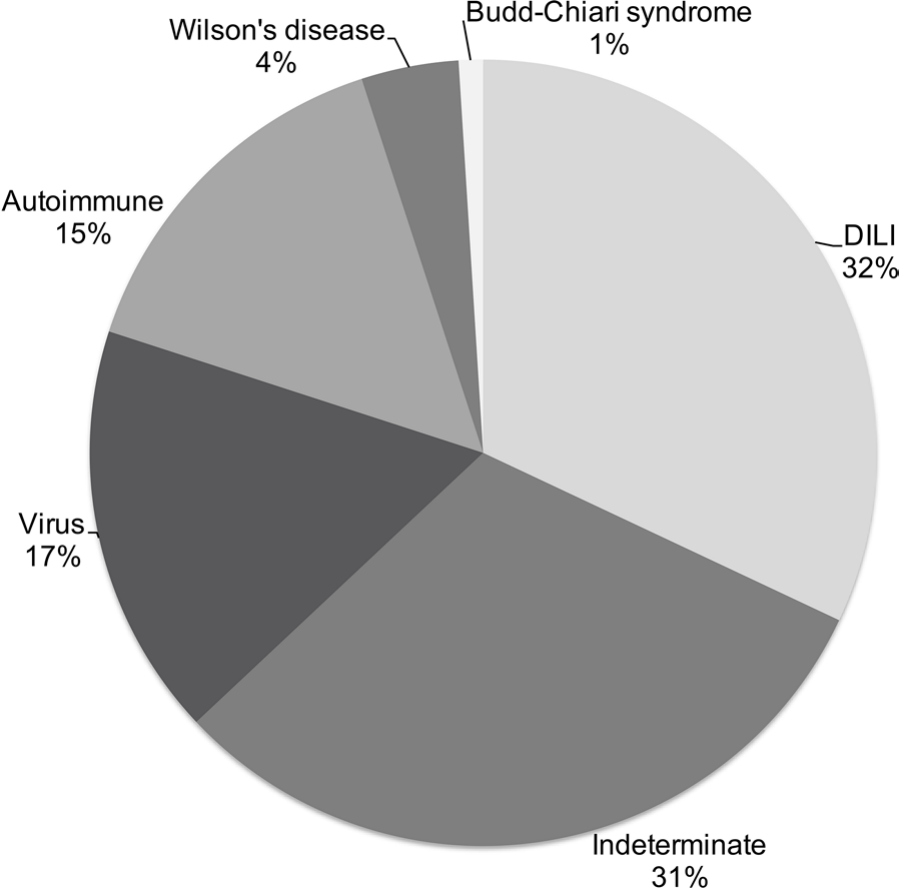
Etiologies in 100 patients with acute liver failure prioritized for liver transplantation. DILI, drug induced liver injury

Figure 1 shows the causes of ALF in the 100 patients. Other causes included Wilson disease in 4% and Budd-Chiari syndrome in 1%. The virus etiology was hepatitis A virus in 17.6% (3), hepatitis B virus in 76.5% (13), and hepatitis C virus in 5.9% (1). Autoimmune hepatitis was more frequently observed in the transplanted group (20.3%). Otherwise non-transplanted patients presented a higher incidence of indeterminate hepatitis (45.2%). Other etiologies were similar between groups (Table 1).

### Predictive factors for mortality on the waiting list

The highest patient survival without undergoing transplantation was 7 days. Based on that, five variables (indeterminate etiology, INR, bilirubin, creatinine, and lactate) were included in a multiple logistic regression model. The analysis identified creatinine and lactate as independent prognostic factors (Table 2). A new CL score was developed, as summarized earlier.

**Table 2:** Independent risk factors for mortality during the wait for liver transplantation

Variable	Estimate	OR (95%CI)	*p* value
Intercept	− 2.886 ± 0.577		< 0.001
Creatinine: µmol/L	0.004 ± 0.001	1	0.013
Lactate: mmol/L	0.242 ± 0.066	1.27 (1.11–1.46)	< 0.001

Multiple logistic regression analysis

**Fig. 2:**
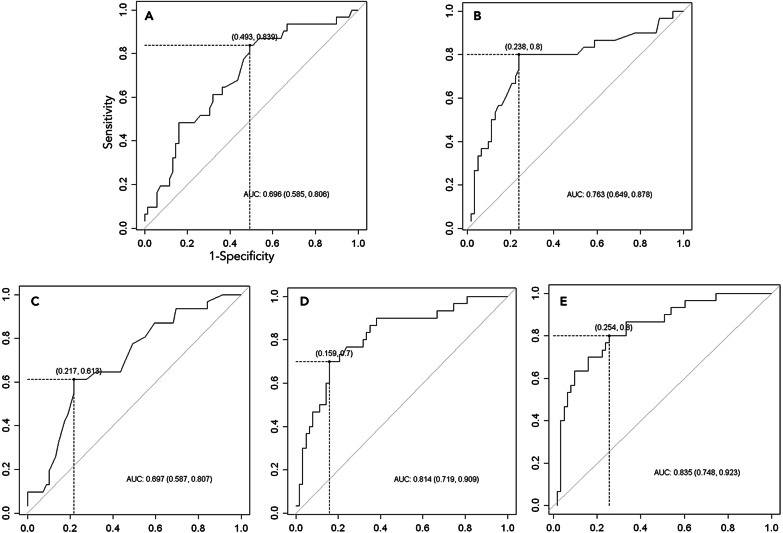
Receiving operator characteristics (ROC) curves for prediction of death in the waiting period for liver transplantation. Analysis of creatinine (**A**), lactate (**B**), MELD score (**C**), BiLE score (**D**) and CL score (**E**)

According to Table 1, the three scores analyzed were discriminators of survival during the waiting period before urgent LT. The new combined CL score showed the highest area under the curve (AUC, 0.835) of the ROC curve (Fig. 2), followed by the BiLE score (0.814), lactate (0.763), MELD score (0.697), and creatinine (0.696). Cutoffs were determined for creatinine (87.52 µmol/L), lactate (5.0 mmol/L), and MELD (44), BiLE (11.02), and CL (179.51) scores, which had sensitivities and specificities of 43% and 88%, 80% and 76%, 61% and 78%, 70% and 84%, and 87% and 71%, respectively. Predictive values are displayed in Table 3.

**Table 3:** Predicting death during the wait for liver transplantation

Variable	Cut-off value	Sensitivity (%)	Specificity (%)	PPV (%)	NPV (%)
Creatinine	87.52 µmol/L	43	88	70	32
Lactate	5.00 mmol/L	80	76	62	89
MELD	44	61	78	56	82
BiLE	11.02	70	84	68	85
Creatinnie-Lactate	179.51	87	71	59	92

*PPV* predictive positive value, *NPV* negative predictive value, *MELD* model for end-stage liver disease, *BiLE* bilirubin lactate etiology

## Discussion

ALF is an acute syndrome with high morbidity, and even cases subjected to urgent LT have inferior survival rates compared to those transplanted electively for chronic liver disease. Several authors have studied prognostic factors for selecting earlier patients who will inevitably have indication for LT [4, 7, 17, 18]. Hence, if patients are listed for highly urgent LT simultaneously, they will be allocated in only chronological order, disregarding the severity of each case.

To date, no criteria have been defined to stratify these patients according to disease severity during the wait for transplantation. Nevertheless, Figorilli et al. [19] evaluated 61 patients diagnosed with APAP-induced ALF and proposed a new score to select patients who are at high risk of immediate postoperative death and futility of LT. Identification of prognostic factors for survival strengthens the discussion of guidelines to optimize the organ allocation for ALF patients. The aim of this study was to evaluate prognostic indicators of death during the wait for urgent LT.

In our results, the similarity in encephalopathy, bilirubin, and INR levels may have occurred in part because all patients selected for urgent transplantation fulfilled KCC. The overall mortality on the waiting list without transplantation was 31%, without any case of spontaneous recovery. The majority of reports have shown cases of spontaneous recovery in groups that have not fulfilled criteria for LT indication [3]. On the contrary, Figorilli et al. [19] observed that 30.1% of 126 patients diagnosed with APAP-induced ALF showed recovery after fulfilling KCC.

In our case series, the more frequent causes of ALF included DILI as the main cause (32%). Although idiosyncratic drugs constitute the dominant cause of hepatitis, in this study, no case of APAP-induced hepatitis was reported. However, APAP is responsible for 13 to 60% of ALF cases in the USA, UK, Nordic countries, and Australia [20, 21]. On the other hand, DILI was reported in 27% of 325 LT cases for ALF, including only 3 cases of APAP toxicity in Brazil [22].

In 2007, we reported 28 cases of DILI, including methyldopa in 25% of the cases, antituberculosis in 14.3%, flutamide in 10.7%, anorexigens in 10.7%, antithyroid agents in 10.7%, and other agents in 28.6% [23]. Patients with indeterminate hepatitis presented a higher mortality of 45%. Other authors have suggested moderate to high mortality ranging from 50 to 97% without LT among patients with autoimmune ALF [20, 24, 25]. Indeterminate etiology is reported in 12–47% of cases of ALF [3, 20]. It is also related to worse prognosis and is included in the KCC and BiLE score as an indicator of worse outcome.

In this study, we suggested possible poorer outcomes of ALF patients who are referred for transplantation with increased creatinine and lactate blood levels, MELD scores, BiLE scores, or the new CL score. This indicates that patients with a rapid increase of these parameters should undergo transplant earlier or should be diagnosed as too sick to treat in order to obtain better results. Further studies are necessary to assess the impact of CL score to evaluate long-term outcome of LT for patients with non-APAP ALF.

The negative impact of renal failure on survival of cirrhotic patients is well known. In 2000, Malinchoc et al. [26] showed that log_e_ creatinine is an independent predictor of the survival of cirrhotic patients undergoing a transjugular intrahepatic portosystemic shunt. In ALF, evaluation of blood creatinine is an important issue since creatinine levels may be underestimated due to liver metabolic failure [27]. Acute kidney injury, which was defined by creatinine greater than 1.5 mg/dL, is a risk factor for the spontaneous survival of ALF patients and is associated with increased early and 1-year mortality after LT [28, 29].

The multiple logistic regression analysis of our data reveled creatinine and lactate blood levels at prioritization day were independent prognostic factors of mortality on the waiting list for LT. Other authors have demonstrated the prognostic implications of high lactate blood levels in decreasing survival of ALF patients, particularly in APAP-induced hepatitis [30, 31]. In 2010, Bernal [32] advised that lactate levels can support decision-making to define the prognosis of ALF patients. Earlier, in a cohort of 48 patients, MacQuillan et al. [33] showed that lactate level at 12 h after patient admission is an independent indicator of survival for both APAP overdose and non-APAP ALF. Our study evaluated only non-APAP induced hepatitis.

The combined CL score was developed to determine the prognosis of patients with non-APAP ALF on the waiting list. This score presented the highest AUC-ROC of 0.835. The cutoff value of 179.51 showed high sensitivity to predict the wait-list mortality with slightly lower sensitivity than the MELD score. In 2002, the system for liver allocation in the US was based on the MELD score [34]. Since then, the MELD-based liver allocation system has been implemented in many centers worldwide [35, 36]. Although the MELD was designed to allocate patients on the waiting list according to cirrhosis severity, during the last decade, the score has been evaluated to determine the prognosis in cases of ALF [37, 38].

In 2004, Kremers et al. [11] evaluated 312 patients with non-APAP ALF and revealed that the MELD score is a predictor of mortality when awaiting LT. In contrast, other reports failed to irrefutably prove the consistent superiority of MELD to replace KCC for predicting outcomes in ALF [39, 40]. A recent meta-analysis including 23 studies revealed similarity in AUC-ROC between MELD (0.78) and KCC (0.76) [41].

In 2008, the BiLE score, including bilirubin levels, lactate levels, and etiology, was proposed to determine the prognosis of patients with ALF exclusively. Evaluation of AUC-ROC suggested that even the BiLE score (0.814) presented greater accuracy than MELD (0.697). Additionally, the sensitivity was higher than that of the MELD score and even KCC, which showed comparable specificity to the BiLE score [17]. On the other hand, Bernal et al. [42] found lower accuracy, sensitivity, and specificity of the BiLE score than KCC after a retrospective evaluation of 422 patients, suggesting other studies should be performed to validate this score.

In 2012, Hadem et al. [43] reinforced that the BiLE score was not proven to have advantages over KCC. Up to now, no other study has been performed to evaluate the BiLE score. However, the present study on 100 patients with non-APAP ALF that fulfilled KCC suggested greater accuracy using the AUC of the ROC curve of the BiLE (0.814) and CL (0.835) scores compared to MELD (0.697) and isolated creatinine (0.696) and lactate (0.763). Additionally, sensitivity and specificity were higher than 70% for the BiLE and CL scores. Unfortunately, it was not possible to evaluate the accuracy of KCC in this series since there was no case of spontaneous recovery available to be included in this study.

Finally, several reports have addressed the implication of prognostic markers to improve the indication for LT in patients with ALF [4, 7, 17, 19, 44]. Nevertheless, since the development of KCC in 1989 [4], no other marker has proven to be more useful [41]. Recently, the Liver Advisory Group created UK revised criteria (UKRC) on behalf the NHS to replace the KCC. UKRC includes lactate levels to indicate urgent LT for APAP-ALF [45]. Outstandingly, for APAP-ALF patients, UKRC showed sensitivity and specificity for hospital mortality of 92.3% and 80.4%, respectively [46].

In 2021, Patidar et al. [47] developed a model based on factor V and suggested that it has more accuracy than KCC to predict LT-free survival. Moreover, in this series, we showed high mortality of patients while awaiting LT. It is possible that these patients were already too sick to undergo transplant, or the transplant indication based on KCC could have been too late. Lately, discussion on determining the futility of LT for high-risk patients has risen [13, 19, 48]. Rocha-Filho et al. [49] showed high early postoperative mortality of patients with ALF complicated with increased intracranial pressure. However, in LT, discussion of futility has traditionally been focused on organ shortage [13]. Currently, attempts to develop accurate definitions of futility have failed, and thus, the recognition of patients who are too sick to treat relies on medical teams.

## Conclusion

In conclusion, our study has shown that creatinine and lactate are independent prognostic factors for the mortality of patients with non-APAP ALF who fulfill KCC. In these patients, CL and BiLE scores can predict mortality with more accuracy than MELD during the prioritization period. Additionally, development of new scores could allow better selection of patients according to the severity of the disease. This would allow for either earlier transplantation of cases at risk of death without LT or the precise identification of cases at high risk of death after transplantation.

## Data Availability

The datasets used and/or analyzed during the current study are available from the corresponding author on reasonable request.

## Abbreviations

ALF: Acute liver failure
LT: Liver transplantation
KCC: King’s College Criteria
MELD: Model for end-stage liver disease
INR: International normalized ratio of prothrombin
ALT: Alanine aminotransferase
AST: Aspartate aminotransferase
BiLE: Bilirubin-lactate-etiology
CL: Creatinine-lactate
DILI: Drug-induced liver injury
APAP: Acetaminophen
AUC: Area under the curve
UKRC: UK revised criteria
